# In Vivo Wound‐Healing and Molecular Docking Studies Support the Traditional Use of *Arisarum vulgare* Aqueous Extract

**DOI:** 10.1002/ptr.70087

**Published:** 2025-09-03

**Authors:** Zineb Bouafia, Amel Boudjelal, Souhila Bouaziz‐Terrachet, Antonella Smeriglio, Mustapha Mounir Bouhenna, Ilyas Yıldız, Ibrahim Demirtas, Domenico Trombetta

**Affiliations:** ^1^ Department of Microbiology and Biochemistry, Faculty of Sciences University of M'sila M'sila Algeria; ^2^ Laboratory of Biology: Applications in Health and Environment University of M'sila M'sila Algeria; ^3^ Laboratory of Applied Chemistry and Materials University of M'hamed Bougara Boumerdes Algeria; ^4^ Laboratory of Theoretical Physico‐Chemistry and Computer Chemistry, Faculty of Chemistry USTHB Algiers Algeria; ^5^ Department of Chemical, Biological, Pharmaceutical and Environmental Sciences University of Messina Messina Italy; ^6^ Centre de Recherche Scientifique et Technique en Analyses Physico‐Chimiques (CRAPC) Bou‐Ismail Algeria; ^7^ Foundation of the Faculty of Health Sciences, Nutrition and Dietetics Department Igdır University Igidir Turkey; ^8^ Department of Biochemistry, Faculty of Science and Arts Igdir University Igdir Turkey; ^9^ Department of Pharmaceutical Chemistry, Faculty of Pharmacy Ondokuz Mayıs University Samsun Turkey

**Keywords:** *Arisarum vulgare* O. Targ. Tozz, in silico molecular docking studies, in vivo wound healing activity, LC‐ESI‐MS/MS analysis, polyphenols, traditional medicine

## Abstract

In Algerian traditional medicine, *Arisarum vulgare* O. Targ. Tozz. (Araceae), locally known as “Elbgouga,” is widely used to treat eczema, wounds, and burns. The aim of this study was to investigate, for the first time and by using in vivo and in silico molecular docking techniques, the possible effects of *A. vulgare* ultrasound‐assisted aqueous extract (AVAE) on wound healing. The phytochemical profile was elucidated by LC‐ESI‐MS/MS analysis. Wistar albino rats were used to evaluate the AVAE ointment's acute cutaneous toxicity and wound‐healing potential (1%, 2%, and 5% AVAEO). Through in silico investigations, TNF‐α, IL‐1β, MMP‐9, TGF‐β, VEGF, and EGFR were examined as possible therapeutic targets. Twenty‐seven phytochemicals, belonging mainly to the flavonoids and phenolic acids' class, were identified and semi‐quantified. The 5% AVAEO‐treated group showed a significantly greater (*p* < 0.001) wound contraction (8–20 days) with respect to untreated and petroleum jelly groups, whereas no statistically significant difference was observed with respect to the Madecassol‐treated group. On the contrary, the two lower doses (1% and 2% AVAEO) showed no statistically significant effects. Docking studies showed that *A. vulgare* bioactive compounds may have therapeutic effects on wound healing by targeting with high affinity TNFα, IL‐1β, MMP‐9, TGF‐βR1, VEGF, and EGFR, counteracting inflammation, angiogenesis, and oxidative unbalance, and promoting wound repair. This study demonstrated that AVAE possesses in vivo wound healing properties and no dermal toxicity, shedding light also on the potential therapeutic targets involved.

## Graphical abstract

Arisarum vulgare aqueous extract showed safe and effective wound‐healing activity in vivo. Molecular docking supported the role of its phytoconstituents in modulating inflammation, angiogenesis and tissue regeneration, validating its traditional use in Algeria.

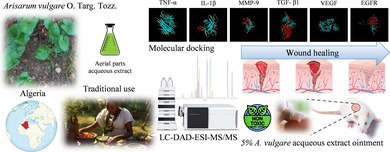

## 1 Introduction

The healing of the skin wounds is a fascinating biological mechanism that has served mammals well throughout history (Tiwari and Pathak 2023). Ignored skin damage can significantly lower quality of life and lead to unintended health issues (Tamri et al. 2014).

The wound healing organization defines the wounds as physical injuries that cause an opening or break in the skin, disrupting normal skin function and structure (Cedillo‐Cortezano et al. 2024). A wound is a damaged tissue condition brought on by physical, chemical, microbiological, or immunological assaults; it is usually linked to a loss of function (Akhtari et al. 2024). Damaged skin can be structurally and functionally restored thanks to a close collaboration between several cell types, pro‐oxidant and pro‐inflammatory mediators, and vascular system; this event involves four often overlapped phases: haemostasis, inflammation, proliferation, and remodeling (Monika et al. 2022). Wounds remain a challenging clinical problem; according to current research, almost six million individuals suffer from chronic wounds globally (Velnar et al. 2009). The necessity for effective wound care is highlighted by the financial burden that reduced productivity and high treatment costs place on the healthcare industry plus the effects on physical and psycho‐social health (Gounden and Singh 2024). Although several synthetic chemical agents are employed in wound healing, they have significant disadvantages, including difficulties and adverse consequences (Najaf et al. 2020). Plant research has received more attention recently worldwide, and a substantial amount of data has accumulated to demonstrate the potential of several traditionally used medicinal herbs (Atanasov et al. 2015). For the treatment of skin‐related issues, almost 80% of people worldwide rely on natural medications (Ekor 2014), and among them, the safe use of medicinal plants has drawn a lot of interest (Hu et al. 2023; Vendrame et al. 2024). They are abundant in bioactive substances, potent molecules that have proven health benefits, including anti‐inflammatory, anti‐cancer, antibacterial, wound healing, and antioxidant properties, and which show promise as therapeutics (Genc et al. 2019; Marques et al. 2023). The crosstalk between the several cells leads to the release of several cytokines, growth factors, and collagen, necessary for the successful wound repair (Pillai et al. 2010). It has been proven that certain phytoconstituents may positively interfere with one or more steps of the wound healing event (Borsaikia et al. 2021). The diversity of bioactive chemicals and their mode of action, alone or combined, like occurs naturally in a plant‐complex, presents a challenge for today's pharmacology. Searching for plants that have been used in worldwide traditional medicine, investigating their phytochemistry, and elucidating if and how their bioactive components may contribute to the observed pharmacological activities is a fascinating endeavour (Wink 2015). This empirical knowledge about plants and their therapeutic properties it is often passed down through oral traditions (Boudjelal et al. 2013). By rigorously studying the efficacy and safety of traditional remedies, researchers can validate centuries‐old practices and integrate them into modern healthcare systems. From this perspective, *Arisarum vulgare* O. Targ. Tozz. was chosen as the plant species to investigate based on two criteria. First, there is a lack of research in Algeria focusing on this plant. Secondly, this plant possesses ethnopharmacological data suggesting its traditional application in treating a multitude of medical conditions, including pain, infections, inflammation, eczema, skin cancer, wounds, and burns (Kadri et al. 2013; Meddour and Meddour‐Sahar 2015; Aydin et al. 2017; Senouci et al. 2023). Considering the positive therapeutic benefits of this plant and its extensive traditional practice for wound therapy, the current study aims to evaluate the safety and efficacy of *A. vulgare* aqueous extract on the healing of excisional wounds by an in vivo investigation. After a careful phytochemical characterization by LC‐ESI‐MS/MS analysis, in silico molecular docking experiments aimed to investigate the interaction of the main phytoconstituents with the essential proteins involved in the wound healing process, such as tumour necrosis factor alpha (TNF‐α), interleukin (IL)‐1β, IL‐1β, matrix metalloproteinases‐9 (MMP‐9), transforming growth factor beta receptor type‐1 (TGF‐βR1), vascular endothelial growth factor (VEGF), and epidermal growth factor receptor (EGFR), were carried out. Indeed, molecular docking studies can be a powerful tool for clarifying the potential connections between plant chemicals and molecular cited targets implicated in the wound healing event (Larouche et al. 2018; Khotimah et al. 2021). Furthermore, this multi‐disciplinary approach may be extremely beneficial for rational medication design and the discovery of potential medicinal agents (Ud‐Din and Bayat 2017; Aly et al. 2023).

## 2 Materials and Methods

### 2.1 Chemicals

Ultra‐pure methanol, ammonium formate, formic acid, and hexane were purchased from Merck (Darmstadt, Germany). High‐purity (≥ 90%) standards of protocatechuic acid, epigallocatechin, chlorogenic acid, hydroxybenzaldehyde, vanillic acid, caffeic acid, caffeine, vanillin, trans‐ferulic acid, o‐coumaric acid, salicylic acid, isoquercitrin, sinapic acid, hesperidin, scutellarin, kaempferol‐3‐glucoside, fisetin, trans‐cinnamic acid, quercetin, protocatechuic ethyl ester, naringenin, kaempferol, luteolin, biochanin A, baicalin, chrysin, and morin were purchased from Sigma Aldrich (Steinheim, Germany). Quercetin‐3‐xyloside was kindly provided by Gaziosmanpaşa University (Turkey).

### 2.2 Plant Material

**FIGURE 1:**
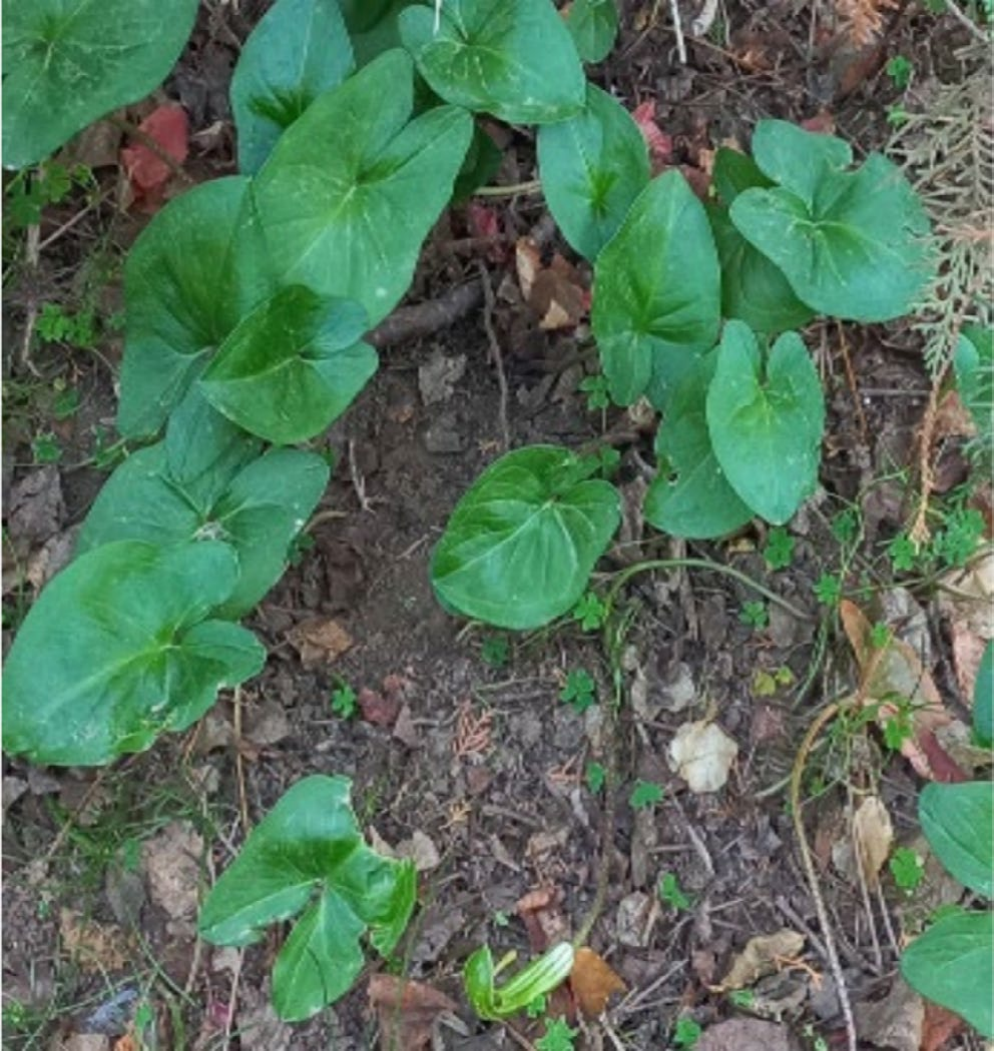
*A. vulgare* (Bouira, Algeria).

*A. vulgare* aerial parts were collected in Bouira province, in the northern region of Algeria (36°23′49″ N and 4°14′55″ E). Taxonomic identification was confirmed by Pr. H. Hadj‐Arab (Faculty of Biological Sciences, LBPO, USTHB, Algeria). A voucher specimen (AB‐110) was collected in the herbarium of the Mohamed Boudiaf University of M'Sila (Algeria). The aerial parts of the plant were dried, powdered by a blade mill (Sayona Electric, France), and kept at room temperature (Figure 1).

### 2.3 Extract Preparation

According to Comlekcioglu et al. (2021), an ultrasound‐assisted extraction was carried out. The powder (50 g) was mixed with 500 mL of deionized water and sonicated (220 W and 37 kHz) for 1 h at room temperature, in an Ultrasonic Water Bath (Elma EL P070H Digital Ultrasonic Baths, Germany). The mixture was then centrifuged at 3500 rpm for 15 min and the supernatants freeze‐dried (Alpha 1–2 LSCbasic, Martin Christ, Germany). The resulting extract (AVAE, extraction yield 34.2% ± 0.48% wt/wt) was stored in an amber‐sealed vial with nitrogen headspace until further analysis (Comlekcioglu et al. 2021).

### 2.4 Phytochemical Characterization

The AVAE phytochemical composition was investigated by LC‐ESI‐MS/MS analysis (Lekmine et al. 2023) using a 6460 Triple Quad LC/MS instrument (Agilent Technologies, Santa Clara, CA, USA). The separation was conducted on a reversed‐phase Poroshell 120 EC‐C18 100 mm × 3.0 mm, 2.7 μm analytical column (Agilent Technologies, Santa Clara, CA, USA), by using a mobile phase consisting of 5.0 mM ammonium formate and 0.1% formic acid in water (solvent A) and 5.0 mM ammonium formate and 0.1% formic acid in methanol (Solvent B) according to the following elution program: 0 to 3 min, 75% A; 3 to 15 min, 50% A; 15 to 16 min, 10% A; 16 to 21 min, 10% A; 21 to 24 min, 75% A. The flow rate was set at 0.4 mL/min, whereas the temperature and injection volume were at 40°C and 5 μL, respectively. The electrospray ionization source (ESI) was used both in negative and positive ionization mode. The mass spectrometry conditions were the following: capillary temperature 350°C, nebulizer (N_2_) gas flow rate 8 L/min, fragmentation voltage 4 kV. The phytochemical components were properly identified and quantified using the multiple reaction monitoring (MRM) technique. The method was validated in compliance with current international guidelines (ICH 1996), evaluating parameters such as linearity, recovery, as well as the limits of detection (LOD) and quantitation (LOQ). The LOD and LOQ values were determined using the approach based on the standard deviation of the response and the slope of the corresponding calibration curves, as recommended by the cited guidelines (ICH 1996).

### 2.5 In Vivo Assay

#### 2.5.1 Animals

Wistar albino rats weighing between 180 and 210 g were used in the studies on acute cutaneous toxicity and wound healing. The Pasteur Institute (Algiers, Algeria) supplied the healthy animals, which were then housed in polypropylene cages under conventional circumstances (12 h light/dark cycle, 23°C, water and feed *ad libitum*). The experiments were carried out according to Directive 2010/63/EU (http://data.europa.eu/eli/dir/2010/63/oj). The experimental protocol was approved by the Ethical Committee of Animal Experimentation (University of Science and Technology Houari Boumediene, Algeria) on September 2, 2023 (approval reference CEEA‐USTHB‐01‐2023/11111) with the following project reference code D01N01UN28012020000.

#### 2.5.2 Ointment Preparation

AVAE was added to the melted petroleum jelly (PJ, Unilever, France) to create ointments with AVAE concentrations of 1%, 2%, and 5% (1%–5% AVAEO), that were stored at +4°C until use (Nwala et al. 2013). The reference treatment was the 1% hydrocotyle Madecassol (Bayer Healthcare, France).

#### 2.5.3 Acute Dermal Irritation

To assess the AVAEO's safety, the examination for acute skin irritation was conducted. For this purpose, the formulation containing the highest AVAE concentration (5% AVAEO), according to the Organisation for Economic Cooperation and Development guideline 404 (OECD 2015), was used. After ointment application on the shaven dorsal fur (50 mm^2^) of the rat, skin responses were evaluated by visual observations to identify any erythema and oedema signs. These reactions were assessed within the first 4 h, and then once a day for the next 2 weeks.

#### 2.5.4 Wound Model and Experimental Animal Groups

After being weighed, the animals were randomly divided into six groups:

Group 1: UT, untreated rats;

Group 2: PJ, rats treated with PJ;

Group 3: MAD, rats treated with Madecassol;

Group 4: 1% AVAEO, rats treated with 1% AVAEO;

Group 5: 2% AVAEO, rats treated with 2% AVAEO;

Group 6: 5% AVAEO, rats treated with 5% AVAEO;

After shaving, the rats were observed for 1 day to confirm the absence of any irritation. Afterwards, animals were anesthetized using ketamine (90 mg/kg) and xylazine (10 mg/kg) (Mashreghi et al. 2013). A 2.5 cm circular wound was made on the lumbar area by a biopsy punch for veterinary use (S.P. Enterprise, Kolkata) and 0.5 g of the different treatments (1%–5% AVAEO, Madecassol, and PJ) were topically applied every day until the wounds' closure (Chabane et al. 2021).

#### 2.5.5 Macroscopic Assessment

The wounds were photographed every 4 days, calculating the contraction's percentage according to the following formula (Boudjelal et al. 2022):

$$\mathrm{WC}\%=\left[\frac{\mathrm{IWS}-\text{SDWS}}{\mathrm{IWS}}\right]x\;100$$

where WC is the wound' contraction, IWS is the initial wound' size, and SDWS is the specific day of the wound' size.

#### 2.5.6 Histological Assessment

At the end of the experiment, the rats were euthanized, and the scar tissues were recovered by a biopsy punch for histological investigations. Tissues were fixed in 10% formalin, dehydrated in ethanol, clarified in toluene, and incorporated in paraffin. Thereafter, 5 μm‐thick tissue sections were observed by light microscopy (Optika B‐500BPL, OPTIKA Srl, BG, Italy) (Marque 2010) after hematoxylin–eosin (H&E) staining.

### 2.6 In Silico Study

A direct molecular docking simulation was performed on six target proteins active sites to explain all potential binding interactions of AVAE phytoconstituents. Protein structures were acquired from Research Collaboratory for Structural Bioinformatics (RCSB). Each protein is defined by its respective Protein Data Bank (PDB) ID and resolution: TNF‐α (PDB code: 2AZ5, resolution 2.10 Å) (He et al. 2005), IL‐1β (PDB code: 6Y8M, resolution 1.90 Å) (Garlanda et al. 2013), MMP‐9 active form (PDB code: 1GKC, resolution 2.30 Å) (Rowsell et al. 2002), TGF‐βR1 (PDB code: 6B8Y, resolution 1.65 Å) (Massagué 2012), VEGF (PDB code: 1FLT, resolution 1.70 Å) (Wiesmann et al. 1997) and EGFR (PDB code: 1 M17, resolution 2.60 Å) (Stamos et al. 2002).

Prior to performing docking calculations, ions, ligands, and water molecules were extracted from each protein crystal structure. Charges and polar hydrogen atoms were then introduced.

The outcome dock‐prepared proteins were used for the subsequent docking process (Bouaziz‐Terrachet et al. 2013).

**FIGURE 2:**
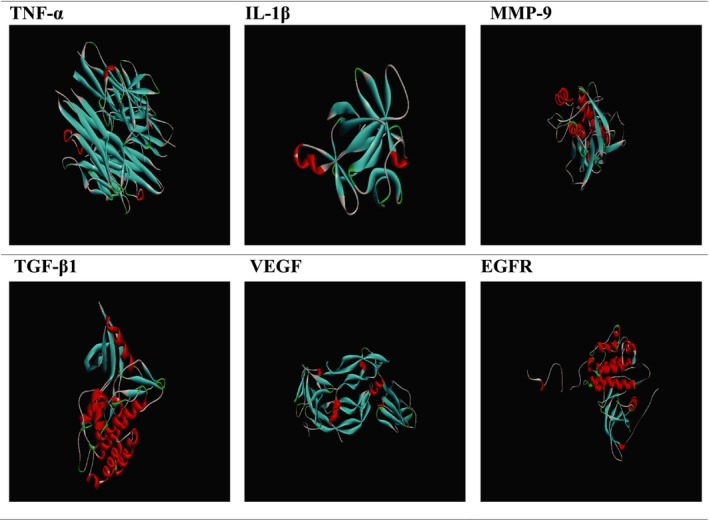
Structure of receptor proteins.

The Discovery Studio program (BIOVIA 2024) was used to include missing residues and hydrogen atoms (BIOVIA 2024). Additionally, the two‐dimensional structures of the phytoconstituents were drawn using Discovery Studio software, where geometry optimizations were performed. Each chemical's molecular design was optimized and used as input for the docking simulations. The AutoDock Vina program was then employed to dock all compounds into the binding pocket of the selected proteins (Figure 2) (Eberhardt et al. 2021).

Directed docking was performed with grid boxes centered around the binding sites of each protein, with specific X, Y, and Z. The dimensions of the grid box were defined to ensure coverage of the entire binding pocket of each protein (Table 1).

**TABLE 1:** Structural properties and grid parameters of selected proteins.

Protein	PDB code	Resolution (Å)	Ligand	Grid box size (Å)	Grid center coordinates (X, Y, Z)
TNF‐α	2AZ5	2.10	307	70.01 × 66.65 × 70.48	(−13.691, 71.60, 26.99)
IL‐1β	6Y8M	1.90	SX2	92.63 × 45.99 × 66.28	(0.40, −2.94, 20.41)
MMP‐9	1GKC	2.30	NFH	52.26 × 44.94 × 74.40	(54.56, 21.26, 129.56)
TGF‐β 1	6B8Y	1.65	D0A	48.02 × 64.52 × 54.15	(8.97, 1.10, 12.26)
VEGF	1FLT	1.70	—	92.63 × 45.99 × 66.28	(0.40, −2.94, 20.41)
EGFR	1 M17	2.60	4Q4	134.80 × 66.55 × 51.51	(3.20, 9.85, 59.41)

The evaluation of the docked complexes was conducted by analyzing the docked energy values (in Kcal/mol) and examining binding interactions, including hydrogen bonds, hydrophobic contacts, and π‐π stacking.

The evaluation of the docked complexes was carried out by analyzing the binding interactions of the native ligand when re‐docked into the receptor structure (Saha et al. 2013).

### 2.7 Statistical Analysis

The results, which represent the mean values (*n* = 5) ± standard error of the mean (SEM), were analyzed using GraphPad Prism 8 software. After a data normality check by the Shapiro‐Wilks test, differences between experimental groups were determined by one‐way analysis of variance (ANOVA), followed by Tukey's test. Differences were considered statistically significant at *p* ≤ 0.05.

## 3 Results

### 3.1 Phytochemical Analyses

The *A. vulgare* aqueous extract (AVAE) phytochemical profile was examined by LC‐ESI‐MS/MS analysis. Table 2 displayed the findings of the semi‐quantitative analysis. Twenty‐seven compounds were identified and semi‐quantified in AVAE. The most frequent substances found were caffeic, vanillic and o‐coumaric acid, naringenin, salicylic, trans‐cinnamic, chlorogenic and trans‐ferulic acid, and kaempferol. The most prevalent class of phytochemicals identified in AVAE was phenolic acids, which were followed by flavonoids.

**TABLE 2:** LC‐ESI‐MS/MS analysis of *A. vulgare* ultrasound‐assisted aqueous extract (AVAE). Results were expressed as mg/g of dry extract (DE).

Compound	RT^a^	MI^b^ (m/z)	FI^c^ (m/z)	Ion mode	mg/g DE^d^	Correlation factor (*R* ^2^)	Linear range (μg/mL)	LOQ^e^ (ng/mL)	LOD^f^ (ng/mL)	Recovery (%)
Protocatechuic acid	5.483	153.0	109.0	Negative	0.055	0.9969	15.63–250	10.43	3.16	93.40
Epigallocatechin	6.752	307.0	139.0	Positive	0.002	0.9995	12.50–200	6.90	2.09	96.40
Chlorogenic acid	7.396	353.0	191.0	Negative	0.261	0.9981	31.25–500	38.25	11.59	98.50
Hydroxybenzaldeyde	7.697	121.0	92.0	Negative	0.054	0.9993	15.63–250	16.40	4.97	99.20
Vanillic acid	7.789	167.0	151.8	Negative	1.490	0.9958	1250–20,000	722.83	219.04	92.10
Caffeic acid	7.859	178.9	135.1	Negative	1.977	0.9994	31.25–500	22.84	6.92	98.60
Caffein	8.412	195.0	137.9	Positive	0.005	0.9986	18.75–300	22.47	6.81	98.50
Vanillin	8.631	153.0	125.0	Positive	0.070	0.9949	62.50–1000	48.15	14.59	93.65
O‐coumaric acid	9.401	163.0	119.1	Negative	1.097	0.9981	15.63–500.0	157.31	47.67	100.30
Salicylic acid	9.785	137.0	93.1	Negative	0.438	0.9981	112.50–1800	157.31	47.67	99.85
Trans‐ferulic acid	10.080	193.1	133.9	Negative	0.228	0.9950	31.25–1000	20.20	6.12	98.14
Sinapic acid	10.432	223.1	208.0	Negative	0.051	0.9972	125–2000	6.40	1.94	93.90
Scutellarin	11.031	462.8	286.8	Positive	0.029	0.9978	9.36–300	10.33	3.13	99.60
Protocatechuic ethyl ester	11.692	181.0	107.9	Negative	0.012	0.9996	15.63–1000	48.05	14.56	96.30
Isoquercitrin	11.734	464.9	302.8	Positive	0.020	0.9982	18.75–300	32.80	9.94	99.80
Quercetin‐3‐ksilozid	12.433	432.7	299.5	Negative	0.005	0.9900	125–2000	61.74	18.71	91.20
Kaempferol‐3‐glucoside	13.021	448.8	286.9	Positive	0.010	0.9997	7.81–125	3.83	1.16	98.00
Fisetin	13.318	287.0	137.0	Positive	0.015	0.9954	15.63–250	35.97	10.9	95.00
Baicalin	13.519	271.0	270.9	Positive	0.005	0.9991	15.63–250	1.75	0.53	101.20
Chrysin	14.364	254.9	153.0	Positive	*tr* ^*g*^	0.9989	1.56‐25	0.23	0.07	95.10
Trans‐cinnamic acid	14.104	149.0	131.1	Positive	0.311	0.9999	31.25–500	36.93	11.19	100.00
Quercetin	14.746	300.8	151.0	Negative	0.059	0.9964	27.50–440	15.38	4.66	101.80
Naringenin	14.805	270.9	119.1	Negative	0.751	0.9960	31.25–500	4.52	1.37	95.50
Hesperetin	15.571	300.9	164.0	Negative	0.023	0.9966	31.25–500	0.99	0.3	93.00
Morin	15.685	302.8	153.0	Positive	*tr*	0.9981	1.56–50	0.43	0.13	100.10
Kaempferol	16.759	284.9	116.9	Negative	0.116	0.9997	312.50–10,000	6.17	1.87	102.10
Biochanin A	17.884	284.9	151.9	Positive	*tr*	0.9963	1.56–25	0.50	0.15	101.10

^a^ RT, retention time.

^b^ MI, molecular ion.

^c^ FI, fragment ion.

^d^ DE, dry extract.

^e^ LOD, limit of detection.

^f^ LOQ, limit of quantitation.

^g^ *tr*, traces.

### 3.2 Wound Healing Study

#### 3.2.1 Acute Dermal Toxicity Study

The 5% AVAEO was tested for acute dermal toxicity. The findings revealed no dermal reactions, such as erythema, edema, or signs of inflammation or irritation. This topical formulation's safety was confirmed, as no noticeable changes were observed over the 14‐day observation period for cutaneous toxicity.

In Algeria, *A. vulgare* is widely used in topical herbal preparations at a 5% dose. The findings of the wound‐healing study, expressed as wound contraction percentage, are shown in Table 3.

**TABLE 3:** Effects of the various treatments on the healing process.

Group	Wound contraction percentage (%)				
	4 days	8 days	12 days	16 days	20 days
UT	2.40 ± 0.40	8.08 ± 0.15	26.08 ± 0.53	46.96 ± 0.32	62.40 ± 0.30
PJ	2.32 ± 0.38	10.56 ± 0.27	24.08 ± 0.26	43.96 ± 0.37	60.90 ± 0.19
MAD	2.90 ± 0.34	20.30 ± 0.30***	52.10 ± 0.25***	65.28 ± 0.39***	96.00 ± 0.16***
5% AVAEO	2.64 ± 0.37	15.04 ± 0.37***	40.64 ± 0.43***	77.70 ± 0.19***	98.32 ± 0.43***

*Note*: Values are expressed as mean ± SEM (*n* = 5). ***p < 0.001 versus untreated group (UT).

Abbreviations: 5% AVAEO: 5% *A. vulgare* aqueous extract ointment treated group; MAD: madecassol treated group; PJ: petroleum jelly treated group.

**FIGURE 3:**
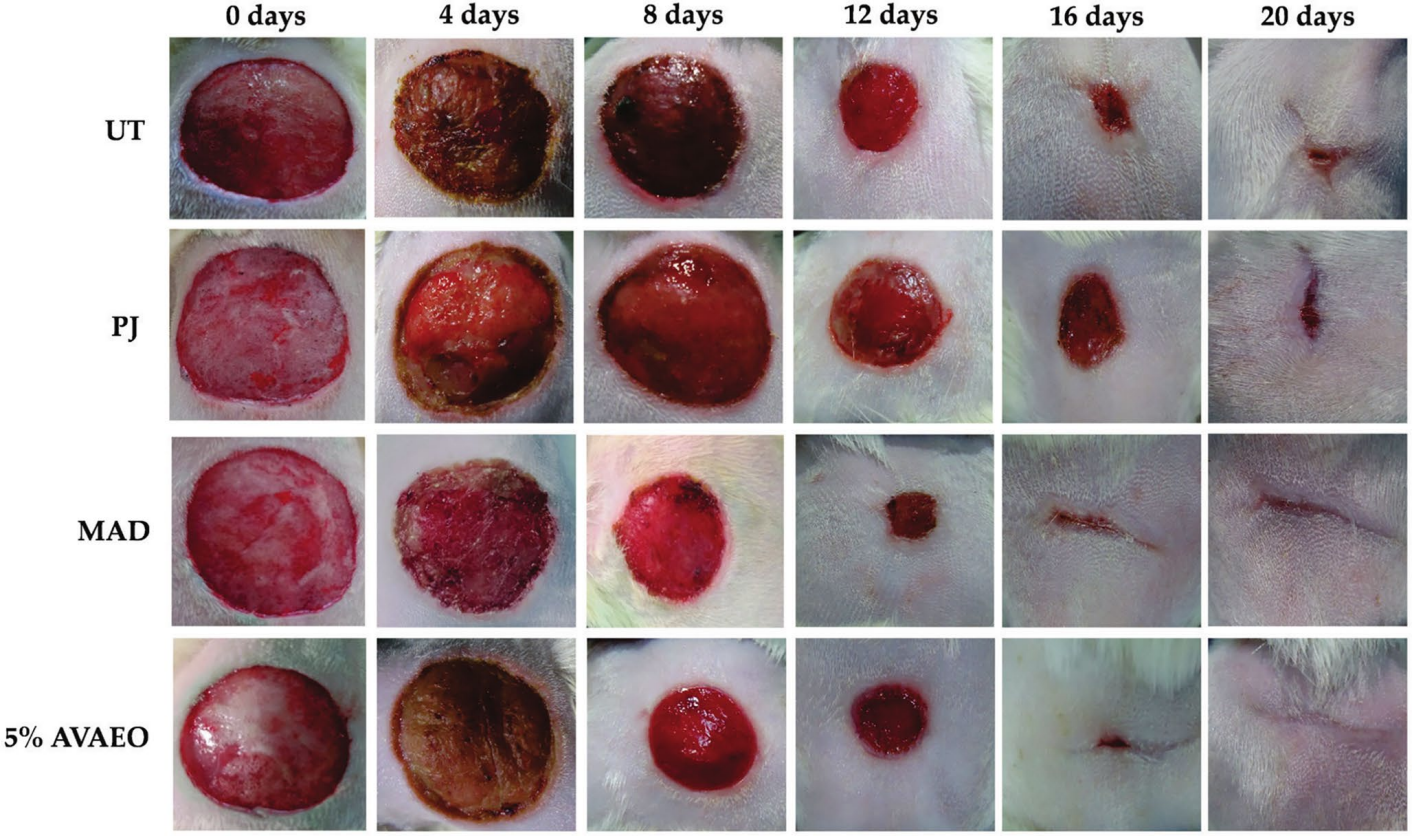
Impact of various treatments on the rat excisional wound model for 20 days. 5% AVAEO: *A. vulgare* aqueous ointment‐treated group; MAD: Madecassol treated group; PJ: petroleum jelly treated group; UT: untreated group.

To investigate the potential dose‐dependent behavior of AVAEO on wound healing, two lower doses (1% and 2% AVAEO) were also examined; however, in both cases, no statistically significant difference was observed with respect to the UT group (data not shown). On the contrary, the maximum dose, which is commonly applied to wounds in Algeria, demonstrated remarkable efficacy. Between 8 and 20 days, a significant (*p* < 0.001) wound contraction was observed in the 5% AVAEO‐treated group in comparison with the UT and PJ groups. Furthermore, there was no discernible difference in wound contraction between the reference drug‐treated group (98.32% ± 0.43% and 96.00% ± 0.16%, respectively) and the 5% AVAEO‐treated group. Furthermore, no significant difference between the PJ‐treated group and the untreated group was recorded during the testing period (Figure 3).

#### 3.2.2 Histological Study

**FIGURE 4:**
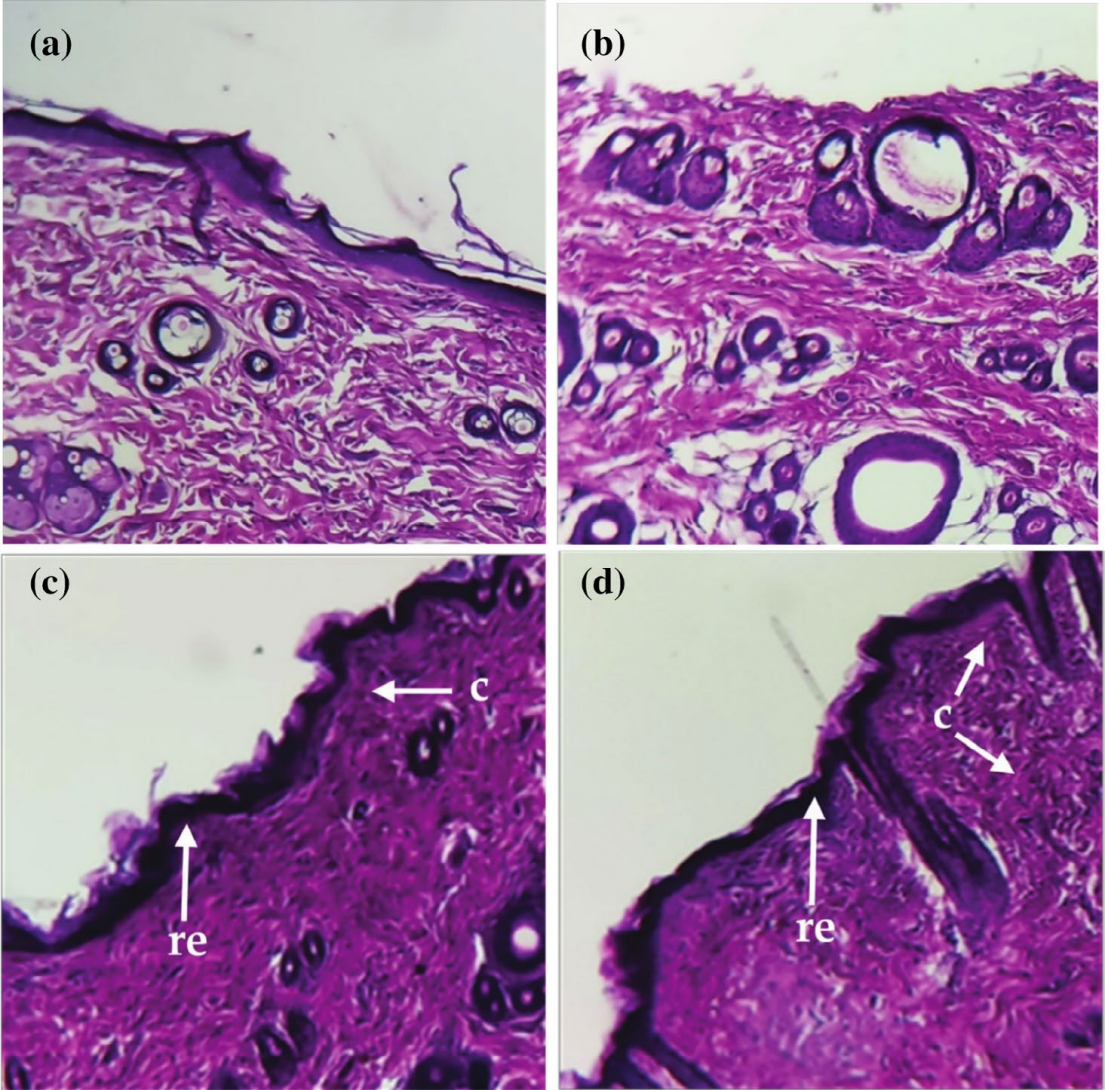
Histological analysis of the rats' excision wounds (20 days) after H&E staining (×40). (a) Untreated group; (b) Petroleum jelly treated group; (c) Madecassol treated group; (d) 5% AVEO treated group. c: collagen fibers; re: re‐epithelialization process.

Figure 4 displays the histopathology study's findings. The tissues treated with MAD and 5% AVAEO (Figure 4c,d) showed better healing and complete re‐epithelialization. Well‐organized collagen bands, granulation tissue, epidermal regeneration, and a higher concentration of fibroblasts were well visible (Figure 4c,d). In contrast, the UT and PJ‐treated tissues (Figure 4a,b) displayed widespread inflammatory foci, incomplete epidermis and dermis cell maturation, as well as fewer collagen fiber depositions and fibroblasts. Furthermore, the MAD and 5% AVAEO groups exhibited higher levels of collagen deposition. This was accompanied by noticeable increases in collagen accumulation and improvements in tissue alignment and maturation within the wound site.

### 3.3 Molecular Docking Results

To examine potential binding interactions and affinities of the discovered drugs, molecular docking was employed (Ashmawy et al. 2019). A full list of 27 phytoconstituents was found and evaluated for their capacity to promote wound healing employing the AVAE LC‐ESI‐MS/MS methodology by examining their binding interactions with the proteins engaging in the multiple phases of the wound healing process (Burgoyne and Morgan 2003; Shady et al. 2022). The binding affinity is represented by the ΔGbinding values, which indicate the strength of the interaction between each phytoconstituent and protein (Khotimah et al. 2021). Lower (more negative) values suggest stronger binding; furthermore, the negative values indicate that the binding process is spontaneous and thermodynamically favorable (Hammad et al. 2020). The evaluation of the docked complexes was conducted by analyzing the lowest energy values (in Kcal/mol) and examining the binding interactions, including hydrogen bonds, Pi‐Alkyl, Pi‐Pi Stacked, Pi‐Pi T‐shaped, Pi‐cation, and van der Waals interactions. These interactions are highly significant in molecular recognition and play a pivotal role in stabilizing the complex: ligand‐protein (Aly et al. 2023; Sachdeo et al. 2024).

#### 3.3.1 Docking With TNF‐α

**FIGURE 5:**
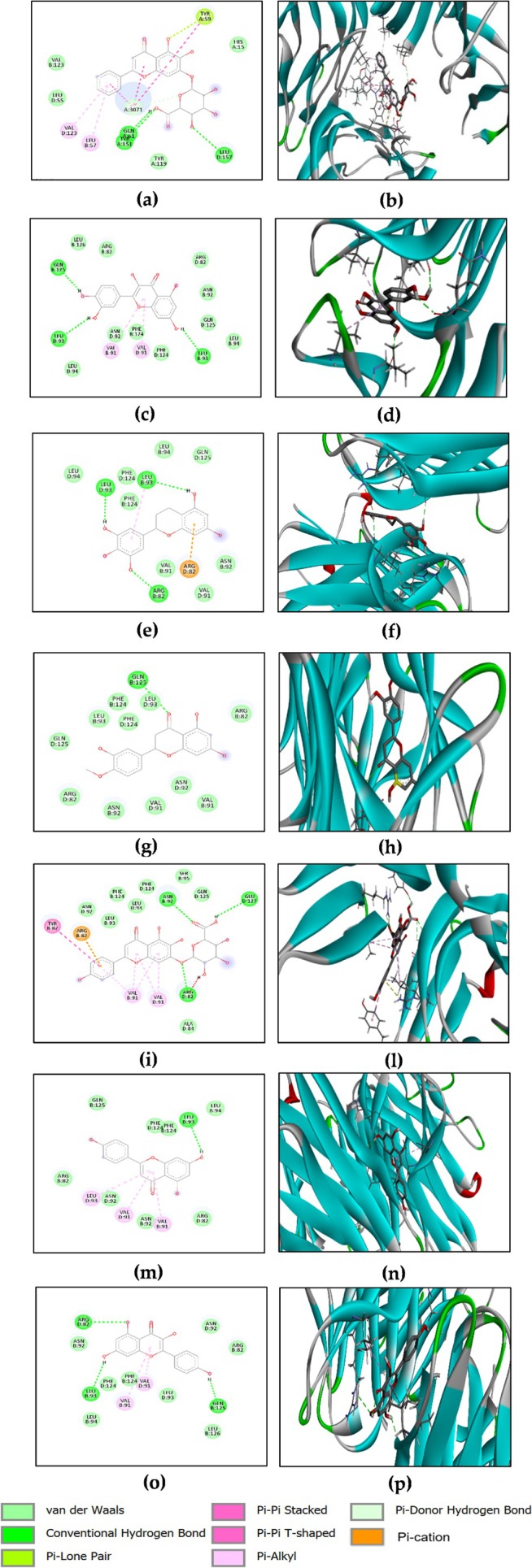
2D and 3D visualizations of the binding interactions of baicalin (a, b), quercetin (c, d), epigallocatechin (e, f), hesperetin (g, h), scutellarin (i, l), naringenin (m, n) and kaempferol (o, p) within the active site of TNF‐α (PDB ID: 2AZ5).

Data shown in Table 4 indicates that none of the investigated compounds surpassed the reference ligand's predicted binding energy value of −9.1 kcal/mol. However, among the compounds tested, epigallocatechin, baicalin, quercetin, hesperetin, scutellarin, naringenin, and kaempferol exhibited the strongest binding within the TNF‐α active site, with energies ranging from −8.7 to −8.1 kcal/mol. Figure 5 reveals the different interactions of these compounds with specific amino acid residues.

**TABLE 4:** Binding energy (ΔGbinding kcal/mol) of different ligands of *A. vulgare* ultrasound‐assisted aqueous extract (AVAE).

Compound	Targeted proteins/ΔG_binding_					
	TNFα 2AZ5	IL1β 6Y8M	MMP9 1GKC	TGFβ1 6B8Y	VEGF 1FLT	EGFR 1 M17
Baicalin	**−8.7**	**−7.6**	**−8.1**	**−9.5**	**−8.8**	**−9.7**
Biochanin A	−7.5	−6.3	−7.6	−8.6	−7.2	−7.8
Cafeic_acid	−6.5	−5.8	−7.1	−8.0	−6.4	−7.0
Caffeine	−7.3	−5.4	−5.8	−7.3	−6.7	−7.5
Chlorogenic_acid	−7.2	−6.4	−7.5	−8.7	−7.1	−7.4
Chrysin	−7.7	−6.7	−7.7	−8.7	−7.0	−8.4
Cinnamic_Acid	−6.3	−5.2	−6.3	−7.4	−6.1	−5.6
Epigallocatechin	**−9.1**	−6.8	−7.2	**−9.6**	−7.4	−8.1
Ferulic_acid	−6.6	−5.2	−6.2	−6.7	−5.7	−5.9
Fisetin	−7.8	−6.6	−7.8	−8.7	−7.4	−8.3
Hesperetin	**−8.4**	**−7.2**	−6.9	**−9.2**	−6.8	−8.4
Hydroxybenzaldehyde	−4.9	−4.4	−5.7	−6.0	−5.0	−5.5
Isoquercetin	−7.6	−6.5	−6.6	−7.1	−6.9	−8.7
Kaempferol	**−8.1**	−6.5	−6.8	**−9.4**	−7.3	−8.2
Kaempferol‐3‐glucoside	−7.3	−6.6	−7.1	−7.9	**−8.1**	−8.5
Morin	−6.9	−6.8	−7.9	−8.1	−7.5	−8.3
Naringenin	**−8.2**	**−7.0**	−7.0	**−9.6**	−7.0	−8.3
O‐coumaric acid	−5.9	−5.1	−6.9	−6.9	−6.1	−5.8
Protocatechuic acid ethyl ester	−5.3	−4.8	−6.1	−7.3	−5.6	−5.8
Protocatechuic acid	−5.6	−4.9	−6.2	−6.3	−5.7	−6.1
Quercetin	**−8.7**	**−7.1**	−7.0	**−9.5**	−7.3	−8.4
Quercetin 3‐xyloside	−7.5	−6.5	−7.0	**−9.4**	−7.2	−8.8
Salicylic Acid	−5.3	−4.7	−6.2	−6.1	−5.6	−5.3
Scutellarin	**−8.3**	**−7.4**	**−8.3**	**−9.1**	**−8.5**	**−9.4**
Sinapic acid	−5.9	−5.0	−5.8	−6.9	−5.4	−6.1
Vanillic acid	−5.2	−5.1	−5.7	−6.9	−5.5	−6.2
Vanillin	−5.0	−4.7	−5.4	−6.5	−5.2	−5.3
Co‐crystallized ligand	−9.1^a^	−5.7^b^	−5.3^c^	−11.5^d^	—	−6.6^e^

*Note*: Bold values indicate the most favorable binding energies.

^a^ 307:6,7‐Dimethyl‐3‐[(Methyl{2‐[Methyl({1‐[3‐(Trifluoromethyl)Phenyl]‐1 h‐Indol3Yl}Methyl)Amino]Ethyl}Amino)Methyl]‐4hChromen‐4‐One.

^b^ SX2: 4‐[(5‐Bromopyridin‐2‐Yl)Amino]‐4‐Oxobutanoic Acid.

^c^ NFH: *N* ~ 2 ~ −[(2R)‐2‐{[Formyl(Hydroxy)Amino]Methyl}‐4‐Methylpentanoyl]‐N,3‐Dimethyl‐L‐Valinamide.

^d^ D0A: N‐(3‐Fluoropyridin‐4‐Yl)‐2‐[6‐(Trifluoromethyl)Pyridin‐2‐Yl]‐7H‐Pyrrolo[2,3‐D]Pyrimidin‐4‐Amine.

^e^ AQ4: [6,7‐Bis(2‐Methoxy‐Ethoxy) Quinazoline‐4‐Yl]‐(3‐Ethynylphenyl)Amine.

Baicalin forms conventional hydrogen bonds with LEU157 (D), GLN61 (A), TYR151 (A), Pi‐Alkyl interactions with LEU57 (A), VAL123 (A), and van der Waals interactions with VAL123 (B), LEU55 (D), HIS15 (A), TYR119 (A); (Figure 5a,b). Quercetin forms conventional hydrogen bonds with GLN125 (B), LEU93 (D), and LEU93 (B). This compound interacts through Pi‐Pi Akyle with VAL92 (D) and VAL91 (B), and van der Waals interactions with many active site residues of the protein including ARG82 (B), LEU126 (B), LEU94 (D), ASN92 (D), PHE124 (B), PHE124 (D), and LEU94 (B) (Figure 5c,d). Epigallocatechin binds via conventional hydrogen bonds with LEU93 (D), LEU93 (B), and ARG82 (B). This compound forms π‐Alkyl interactions with LEU93 (B), and Pi‐cation interaction with ARG82 (B). Also, it forms some van der Waals interactions with many active site residues of the protein including LEU94 (D), PHE124 (D), and VAL91 (D) (Figure 5e,f). Hesperetin engages with GLN125 (B) through hydrogen bond interaction and many van der Waals interactions with residues of the binding site such as PHE124 (B), LEU93 (D), and GLN125 (D) (Figure 5g,h). Scutellarin interacts with ASN92 (B), GLU127 (D), and ARG84 (D) through conventional hydrogen bonds, π‐Alkyl interactions with VAL91 (D), VAL91 (B), Pi‐Pi Stacked, and Pi‐Pi T‐shaped with TYR87 (B). Also, it forms van der Waals interactions with some residues of the active site such as LEU93 (B), ASN92 (D), and PHE124 (D). A Pi‐cation interaction was also observed with ARG82 (B) (Figure 5i,l). Naringenin binds to LEU93 (B) via conventional hydrogen bonds, Pi‐alkyl with VAL91 (D), LEU93 (D), and VAL91 (B). Some van der Waals interactions were observed with residues of the active site such as LEU94 (B), GLN125 (B), and PHE124 (D) (Figure 5m,n). Kaempferol interacts with ARG82 (D), LEU93 (B) and GLN125 (B) through conventional hydrogen bonds, VAL91 (D), and VAL91 (B) through Pi‐alkyl interactions and van der Waals interactions with conserved residues namely PHE124 (B), LEU94 (B) and LEU126 (B) (Figure 5o,p).

#### 3.3.2 Docking With IL‐1β

**FIGURE 6:**
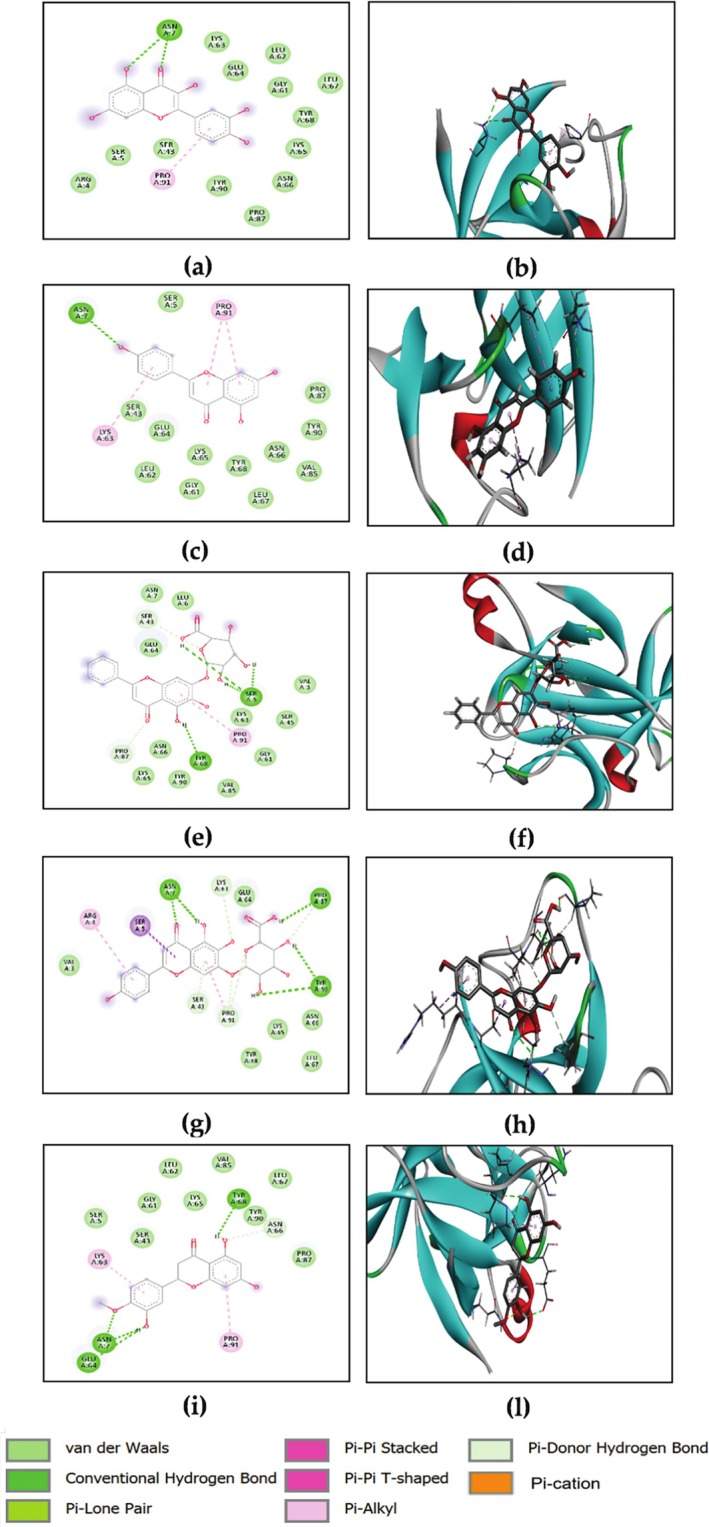
2D and 3D visualizations of the binding interactions of baicalin (a,b), scutellarin (c, d), hesperetin (e, f), quercetin (g, h), and naringenin (i, l) within the active site of IL‐1β (PDB ID: 6Y8M).

The current docking strategy involves the use of baicalin, scutellarin, hesperetin, quercetin, naringenin, epigallocatechin, morin, chrysin, fisetin, and kaempferol. Isoquercetin, kaempferol, quercetin, chlorogenic acid, biochanin A, and caffeic acid showed a noteworthy affinity for IL‐1β, exhibiting a higher binding score contrasted to the co‐crystallized ligand (ΔGbinding = −5.7 kcal/mol), as shown in Table 4. Figure 6 illustrates the interactions of the five components, which showed higher affinity to the docked receptor. The binding of baicalin to IL‐1β occurs through hydrogen bond interactions with TYR 68 (A) and with three SER5 (A); scutellarin interacted with ASN7 (A), PRO87 (A) and TYR90 (A); hesperetin interacted with TYR68 (A), ASN7 (A) and GLU64 (A); quercetin interacted with ASN7 (A) and naringenin interacted with ASN7 (A). The investigated compounds interacted through π–π Akyle with PRO91 (A), LYS63 (A), ARG4 (A) and LYS63 (A). Van der Waals interactions with conserved residues, namely GLU64 (A), TYR68 (A) and ASN66 (A), were also observed.

#### 3.3.3 Docking With MMP‐9

**FIGURE 7:**
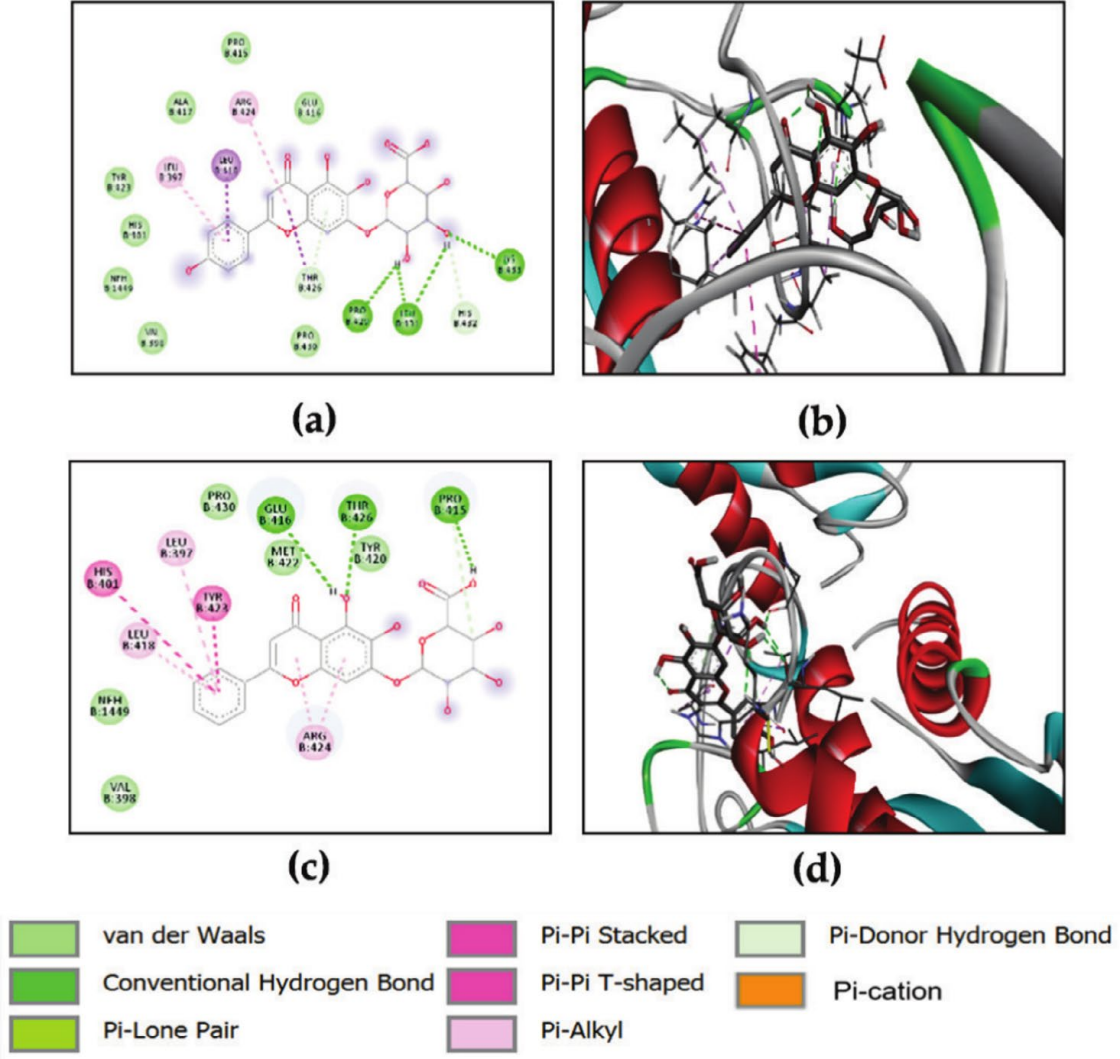
2D and 3D visualizations of the binding interactions of scutellarin and baicalin within the active site of MMP‐9 (PDB ID: 1GKC).

The advancement of MMP‐9 inhibitors has been a promising strategy in the context of wound healing (Khotimah et al. 2021). The ligand energy score was −5.3 kcal/mol. All phytoconstituents revealed a higher affinity showing binding energies ranging from −8.3 to −5.4 kcal/mol (Table 4). Scutellarin and baicalin showed the strongest binding ability, −8.3 and −8.1 kcal/mol, respectively. Figure 7 shows the interactions of scutellarin and baicalin with the MMP‐9 active site. The binding of scutellarin to MMP‐9 occurs through hydrogen bonds with PRO429 (B), LEU431 (B) and LYS433 (B). Baicalin interacted via hydrogen bonds with GLU 416 (B), PRO415 (B) and THR426 (B).

#### 3.3.4 Docking With TGF‐βR1

**FIGURE 8:**
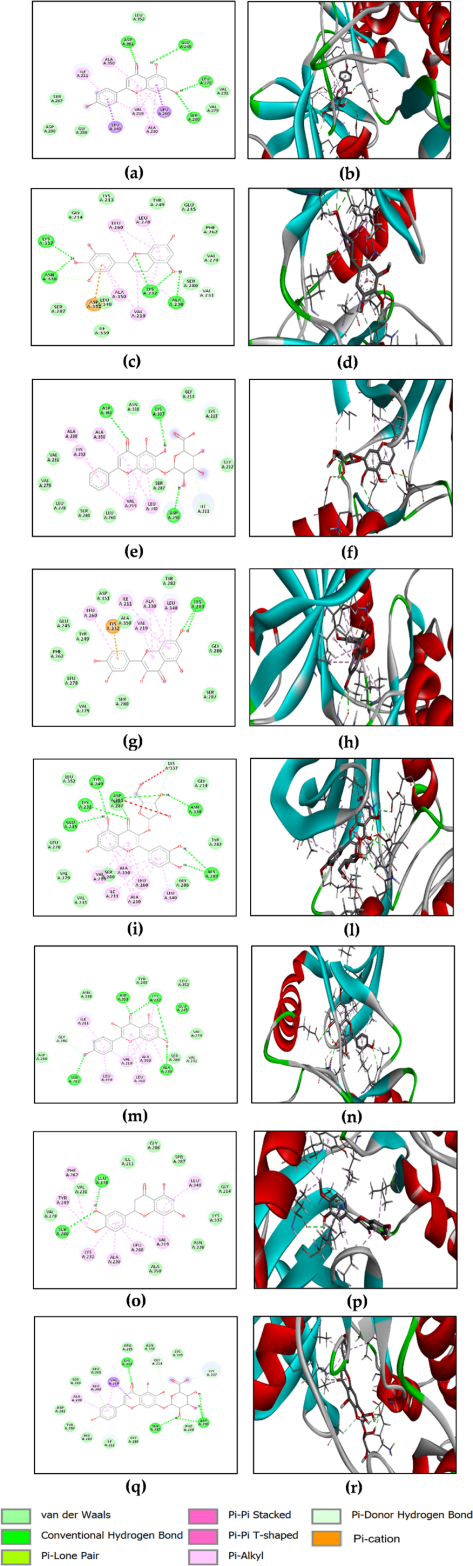
2D and 3D visualizations of the binding interactions of naringenin (a, b), epigallocatechin (c, d), baicalin (e, f), quercetin (g, h), quercetin‐3‐xyloside (i, l), kaempferol (m, n), hesperetin (o, p) and scutellarin (q, r) within the active site of TGF‐βR1 (PDB ID: 6B8Y).

According to Table 4, none of the tested compounds exhibited higher anticipated binding energies than the reference ligand, which had a value of −11.5 kcal/mol. However, all the substances in the list displayed significant binding affinities to TGF‐βR1, with values ranging from −9.60 to −6.0 kcal/mol. Among these compounds, naringenin, epigallocatechin, baicalin, quercetin, quercetin‐3‐xyloside, kaempferol, hesperetin, and scutellarin showed the strongest binding ability, as indicated by their low energy score. Figure 8 shows the binding of naringenin to TGF‐βR1 through hydrogen bonds with ASP351 (A), GLU245 (A), LYS232 (A), LEU278 (A), and SER280 (A). Epigallocatechin is hydrogen bonded with LYS337 (A), ASN338 (A), LYS232 (A), and ALA230 (A). Baicalin is hydrogen bonded with ASP351 (A), LYS337 (A), and ASP290 (A). Quercetin interacted via hydrogen bonding with two HIS283 (A) residues, while quercetin‐3‐xyloside interacted via hydrogen bonding with GLU 245 (A), LYS232 (A), TYR 249 (A), ASP351 (A), ASN338 (A), and HIS283 (A). Kaempferol is hydrogen bonded with GLU245 (A), ASP351 (A), LYS292 (A), ALA230 (A), and SER287 (A). Hesperetin interacted with LEU278 (A) and SER280 (A). Finally, scutellarin interacted via hydrogen bonding with LYS232 (A), ASP290 (A), and SER287 (A).

#### 3.3.5 Docking With VEGF

**FIGURE 9:**
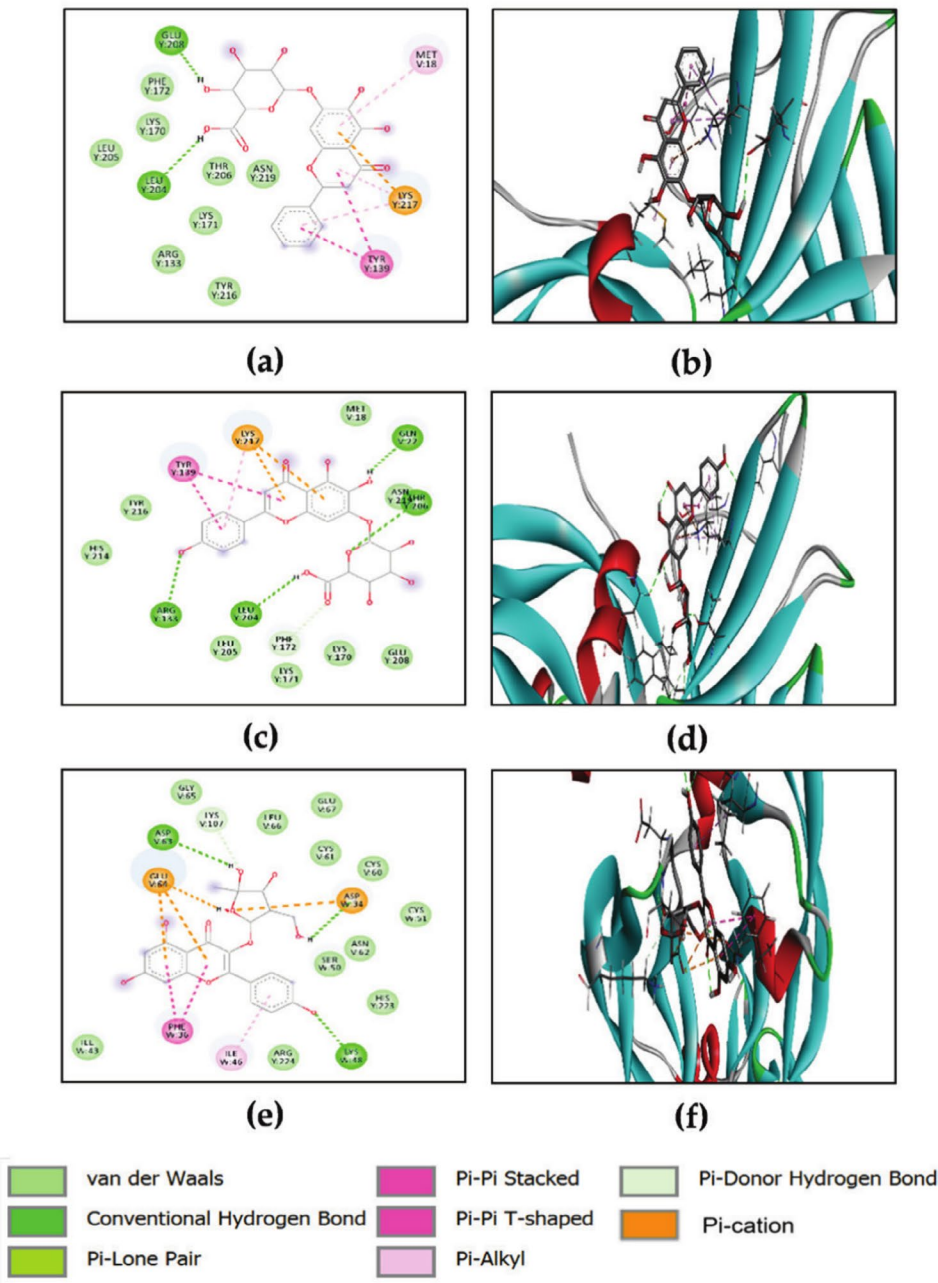
2D and 3D visualizations of the binding interactions of baicalin (a, b), scutellarin (c, d), and kaempferol‐3‐glucoside (e, f) within the active site of VEGF (PDB ID: 1lft).

Docking studies revealed close values of binding energies for several compounds (Table 4). Baicalin, scutellarin, and kaempferol‐3‐glucoside indicated the lowest binding energies among the targeted substances with −8.8, −8.5, and −8.1 kcal/mol, respectively. Figure 9 illustrates the binding of baicalin to VEGF through hydrogen bonds with GLU208 (Y) and LEU204 (Y); scutellarin is hydrogen bonded with GLN22 (V), THR206 (Y), LEU204 (Y), and ARG133 (Y). Kaempferol‐3‐glucoside interacted via hydrogen bonds with ASP63 (V) and LYS48 (W). Other favorable interactions were observed between these compounds and active site residues of the investigated protein, namely Pi‐Pi stacked, Pi‐Pi‐T‐shaped, Pi‐cation, and van der Waals interactions (Figure 9).

#### 3.3.6 Docking With EGFR

**FIGURE 10:**
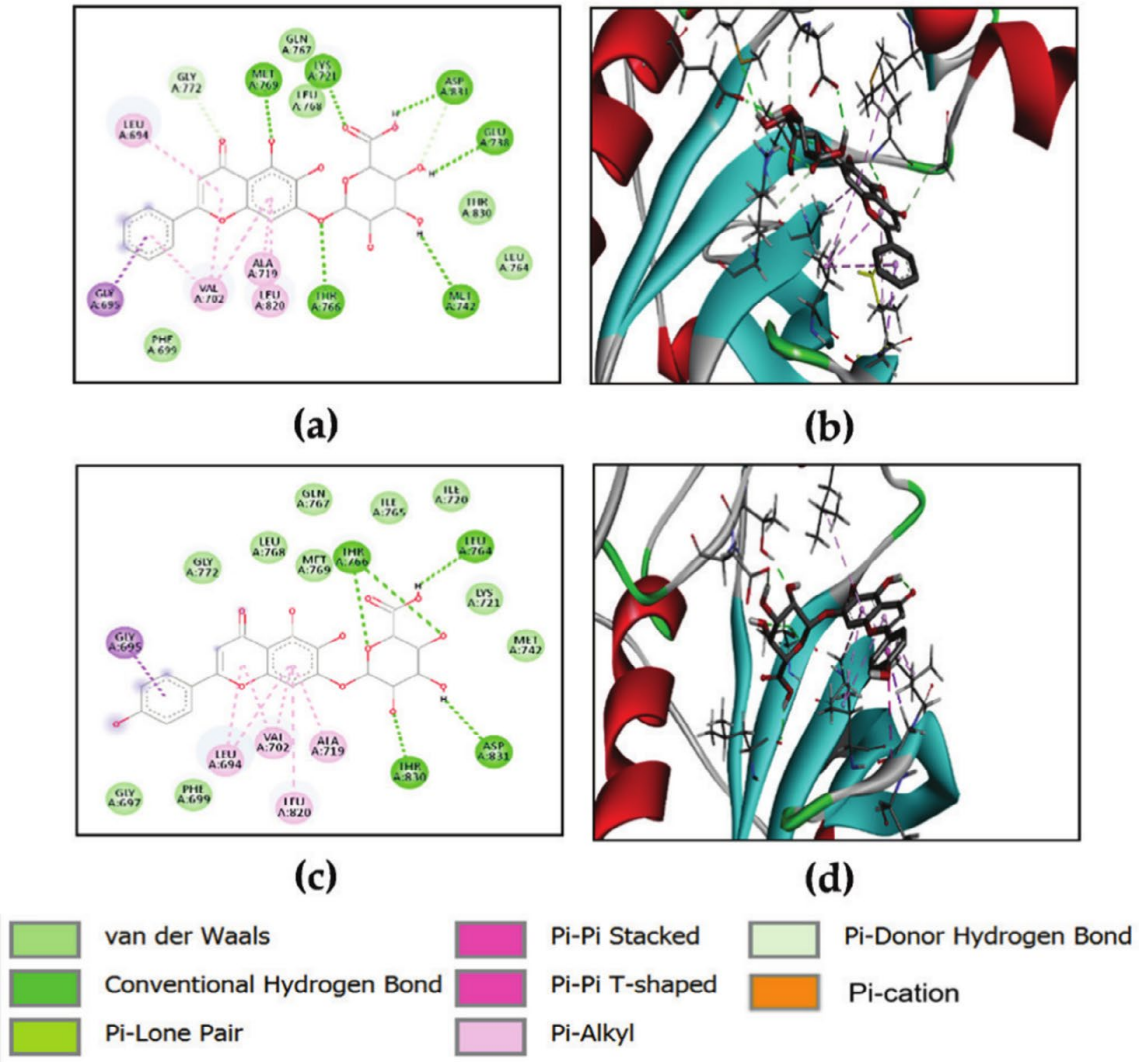
2D and 3D visualizations of the binding interactions of baicalin (a,b) and scutellarin (c,d) within the active site of EGFR.

The current docking strategy involves the use of baicalin, scutellarin, hesperetin, quercetin, naringenin, epigallocatechin, morin, chrysin, fisetin, and kaempferol‐3‐glucoside. The results indicate that isoquercetin, kaempferol, quercetin‐3‐xyloside, chlorogenic acid, biochanin A, and caffeic acid showed a noteworthy affinity for EGFR, exhibiting a higher binding score ranging from −9.7 to −7.0 kcal/mol, compared to the co‐crystallized ligand (ΔGbinding = −6.6 kcal/mol). Baicalin and scutellarin demonstrated the lowest binding energies among the tested compounds. Figure 10 illustrates the binding of baicalin to EGFR through hydrogen bonds with MET769 (A), LYS721 (A), ASP831 (A), GLU738 (A), MET742 (A), and THR766 (A). Scutellarin interacted via hydrogen bonding with THR766 (A), LEU764 (A), THR830 (A), and ASP831 (A). Other favorable interactions were observed between these compounds and active site residues of the investigated protein, namely Pi‐Pi stacked, Pi‐Pi‐T‐shaped, Pi‐alkyl, Pi‐cation, and van der Waals interactions (Figure 10).

## 4 Discussion

The use of ultrasound‐assisted extraction has been acknowledged for its remarkable efficiency in extracting natural compounds. The benefits of this technique include decreased costs and increased efficiency, mainly due to the mechanical impact and acoustic cavitation phenomena caused by ultrasonic waves (Pollini et al. 2020; Bhargava et al. 2021; Mapholi and Goosen 2023; Bontzolis et al. 2024).

This is the first research that investigates the phytochemical profile of the aerial parts of *A. vulgare* by LC‐ESI‐MS/MS analysis, and no data are today available also about *A. vulgare* aerial parts. There is only one study concerning a seed methanol–water (7:3) extract analyzed by RP‐HPLC (Kadri et al. 2013), which identified rutin, gallic acid, and caffeic acid as the main phenolic compounds. Very limited studies have focused on alkaloids in the tubers of this plant species (Melhaoui and Jossang 1992; Melhaoui and Jossang 1995). These findings cannot be discussed by contrasting them with those of other *Arisarum* genus or with those of earlier research on the same plant species.

However, caffeic, p‐coumaric, ferulic, and salicylic acid, as well as vanillin, kaempferol, and quercetin, were already reported in *Arum palaestinum* , another genus belonging to the *Araceae* family (Abu‐Reidah et al. 2015). Most of the detected compounds have been previously documented in the literature for their antioxidant, anti‐inflammatory, and anti‐cancer properties, as well as for the atherosclerosis prevention, hypertension management, antidiabetic effects, and the ability to heal wounds (Wang et al. 2018; Akter et al. 2023).

Due to their antibacterial, antioxidant, and tissue‐regenerative properties, secondary metabolites have been the subject of several studies that have examined their function in protecting the skin and healing various wound types (Casadiego et al. 2023). The bioactivity of phenolic compounds is closely associated with their modes of action and chemical composition (Shetty et al. 2008). The detected compounds include protocatechuic acid, hydroxybenzaldehyde, caffeic acid, vanillin, o‐coumaric acid, salicylic acid, chlorogenic acid, isoquercitrin, kaempferol‐3‐glucoside, naringenin, and hesperidin, all showing various therapeutic effects, reducing oxidative stress, inflammatory response, promoting wound healing and regeneration processes (Sachdeo et al. 2024). Additionally, these molecules exhibit significant improvement in delayed wound healing in rats while also effectively boosting the production of antioxidant enzymes and modulating inflammatory cytokines in the wounds (Venkadakrishnan et al. 2024). In general, oxidative stress slows the healing process of wounds; considering this perspective, antioxidant agents like polyphenols not only protect cells against oxidative damage but also enhance the antioxidant enzymes' action. This dual action can accelerate the therapeutic procedure following a wound incision (Yazarlu et al. 2021). Prior research has shown that herbal treatments work biologically by interacting with several pathways and focusing on many important phases of the mechanism of wound healing. These phases include angiogenesis, deposition of collagen fibers, and re‐epithelialization (Yazarlu et al. 2021). Certain polyphenols have astringent qualities that may promote wound contraction and accelerate re‐epithelialization. They also promote the synthesis of growth factors that aid in the wound‐healing event, including TGF‐β, EGF, and VEGF (Okabe et al. 2013; Firmansyah et al. 2024). The phytochemical composition of AVAE suggests that the significant abundance of flavonoids and phenolic acids may be responsible for the plant's remarkable capacity for healing.

It is well‐known that some medicinal plants can promote angiogenesis, fibroblasts proliferation, and production of provisional extracellular matrix (Vitale et al. 2022). Plant extracts may promote wound closure by targeting key factors participating in the healing process (Fagot et al. 2018). The AVAE’ constituents may positively impact different phases of the healing process for wounds during the appropriate timeframe, resulting in enhanced effectiveness; indeed, these compounds may enhance collagen production, stimulate the generation of new skin cells, facilitate the development of granulation tissue, and increase vascular formation (Mathew‐Steiner et al. 2021).

The creation of synergy, which amplifies the benefits of natural goods and contemporary treatment procedures, is most likely the cause of AVAEO's improved effectiveness. Numerous investigations have shown that these compounds' antibacterial, antioxidant, and anti‐inflammatory properties produce this synergistic interaction (Akhtari et al. 2024).

In the wounded skin regeneration process, inflammation is the most important stage (Velnar et al. 2009). TNF‐α, a crucial multifunctional inflammatory cytokine, is synthesized in large quantities by activated monocytes in cases of inflammation, and it is fundamental in both normal physiological functions and abnormal pathological processes (Boshra et al. 2023). Increased levels of this pro‐inflammatory marker disrupt the fibroblasts' activity, leading to a decrease in collagen, hydroxyproline, and granulation tissue deposition (Dharshan 2018). As a result, wound recovery is impaired. On the other hand, inhibiting the effects of TNF‐α has been demonstrated to reduce inflammation and accelerate the healing of wounds by increasing collagen deposition and matrix formation (Ahmed et al. 2022). Other studies suggest that epigallocatechin has been widely reported to inhibit TNF‐α production; baicalin has shown similar anti‐inflammatory properties by reducing TNF‐α levels, and quercetin has been documented to modulate inflammatory responses by inhibiting TNF‐α production (Zhang et al. 2017; Boots et al. 2020). These results indicate that the phytochemical composition of AVAE could be able to reduce inflammation marked by the overproduction of TNF‐α during skin damage.

Several innate immune system cell types produce the potent pro‐inflammatory cytokine IL‐1β, which encourages inflammation at the site of injury (Boshra et al. 2023). IL‐1β is an important modulator of wound healing, particularly in initiating and regulating the reaction of inflammation. While it plays essential roles in recruiting immune cells, activating fibroblasts, and promoting angiogenesis, its activity must be carefully regulated to prevent chronic inflammation and ensure proper wound healing (Boshra et al. 2023). The docking results suggest that the binding affinities of baicalin, scutellarin, hesperetin, quercetin, naringenin, and other phytoconstituents for IL‐1β align with other literature findings. Baicalin and quercetin have been noted for their significant anti‐inflammatory activities through their interaction with cytokines such as IL‐1β (Dinarello 2011; Zhao et al. 2014).

The enzyme MMP‐9 is essential for wound healing because it facilitates a number of cellular processes and remodels the extracellular matrix (ECM). By breaking down ECM components, MMP‐9 creates pathways that allow cells to migrate to the wound site. This includes the migration of keratinocytes for re‐epithelialization, as well as endothelial cells for new blood vessel formation (Ashames et al. 2024). Proper control of MMP‐9 is essential for timely and effective wound healing. Dysregulation of MMP‐9 activity can contribute to chronic wounds, making it a potential therapeutic target to enhance healing processes (Mathew‐Steiner et al. 2021). According to these results, scutellarin and baicalin have been identified in other studies as having strong binding affinities to MMP‐9 (Li et al. 2013; Gao et al. 2017). Therefore, these phytochemicals have been anticipated to possess the capacity to accelerate the wound healing process.

TGF‐βR1 is a critical receptor in the TGF‐β signalling pathway, influencing significantly various steps of the wound healing process. It regulates cell proliferation, migration, and ECM production, which are crucial for appropriate wound treatment and tissue granulation and remodelling (Kiritsi and Nyström 2018). The control of TGF‐βR1 proteins is crucial for achieving the wound healing process (Velnar et al. 2009; Dharshan 2018). According to the docking data, 16 phytoconstituents may interact with TGF‐βR1; this could be an indicator of their alleged inflammatory characteristics during the healing process of wounds, demonstrating their possible contribution to several wound healing phases (Wang et al. 2006).

VEGF is a signaling protein that plays a key role in wound healing, particularly in the angiogenesis process, granulation tissue production, and wound closure quality. It enhances endothelial cell function, increases vascular permeability, and ensures a sufficient blood supply to the healing tissue (Ahmed et al. 2022; Ashames et al. 2024). Current research has revealed that VEGFRs are also expressed by other cell types, such as keratinocytes and macrophages, which can directly respond to VEGF (Burgoyne and Morgan 2003). Although our docking findings suggest that all phytoconstituents have the capacity to interact with VEGF, which may contribute to their reported angiogenesis properties in the wound healing process, other studies suggested that baicalin and scutellarin exhibited a binding energy of −8.7 kcal/mol when docked with VEGF, which closely aligns with our finding (Li et al. 2014; Lutfiya et al. 2019).

EGFR is a transmembrane protein that, upon binding with its specific ligands such as Epidermal Growth Factor (EGF) and Transforming Growth Factor‐α (TGF‐α), activates several signaling pathways crucial for various cellular processes. It has been shown in several studies that EGFR promotes the healing process in both laboratory animals and humans (Lu et al. 2021; Firmansyah et al. 2024). EGFR plays a key role in the migration of keratinocytes, fibroblast functioning, and granulation tissue development (Zhou et al. 2017). Table 4 highlights the findings of docking research pertaining to the receptors implicated in the proliferation phase.

Our outcomes are consistent with prior studies that have shown the potential of natural compounds in enhancing EGFR‐mediated signaling pathways, supporting the re‐epithelialization and proliferation phases of wound healing (Kadioglu et al. 2015). According to the docking findings, the 17 phytoconstituents have the capacity to interact with EGFR, which may contribute to their reported re‐epithelialization effect in the wound healing process and to the proliferation signaling process onset.

## 5 Conclusions

In conclusion, our research highlights the considerable potential of *A. vulgare* aqueous extract as a therapeutic agent for wound healing. The findings demonstrate that this extract not only promotes effective wound closure but also exhibits no dermal toxicity, supporting its traditional use in Algeria. The identification of 27 phytochemicals by LC‐ESI‐MS/MS analysis suggests that these compounds may act synergistically to enhance the healing process, as further supported by favorable docking scores from in silico studies.

A limitation of the present study is the absence of in vitro validation of the predicted molecular targets. Although molecular docking provided initial insights into possible mechanisms of action, experimental confirmation at the cellular or enzymatic level is necessary to fully substantiate these predictions. These studies will be the subject of a subsequent investigation aimed at providing further mechanistic insight into the biological relevance of the identified metabolites.

Overall, this study provides a valuable foundation for future research aimed at understanding the mechanisms underlying the wound healing properties of *A. vulgare* , and opens perspectives for the development of innovative, nature‐derived therapeutic strategies for the treatment of chronic wounds. Moreover, it contributes meaningful data to the field of natural product‐based wound care, bridging traditional knowledge with modern scientific approaches.

## Author Contributions

**Zineb Bouafia:** formal analysis, investigation, writing – original draft. **Amel Boudjelal:** conceptualization, investigation, visualization, supervision, writing – review and editing. **Souhila Bouaziz‐Terrachet:** investigation, formal analysis, supervision, visualization, writing – review and editing. **Antonella Smeriglio:** data curation, visualization, supervision, writing – review and editing. **Mustapha Mounir Bouhenna:** methodology, investigation, supervision. **Ilyas Yıldız:** conceptualization, methodology, investigation. **Ibrahim Demirtas:** conceptualization, methodology, validation, supervision, investigation. **Domenico Trombetta:** data curation, visualization, supervision, writing – review and editing.

## Conflicts of Interest

The authors declare no conflicts of interest.

## Data Availability

The data that support the findings of this study are available from the corresponding author upon reasonable request.
